# Gastrointestinal Fermentable Polysaccharide Is Beneficial in Alleviating Loperamide-Induced Constipation in Mice

**DOI:** 10.3390/nu15204364

**Published:** 2023-10-13

**Authors:** Buyu Liu, Zhiguo Zhang, Xingquan Liu, Weiwei Hu, Weicheng Wu

**Affiliations:** 1 Food Science Institute, Zhejiang Academy of Agricultural Sciences, Hangzhou 310021, China; lbyuri@outlook.com (B.L.); huww@zaas.ac.cn (W.H.); 2 College of Food and Health, Zhejiang Agriculture and Forestry University, Hangzhou 311300, China; liuxq@zafu.edu.cn

**Keywords:** sweet potato, polysaccharide, constipation, short-chain fatty acids (SCFAs), gut microbiota, fermentation

## Abstract

To investigate the role of gastrointestinal (GI) polysaccharide fermentation in alleviating constipation, two polysaccharide fractions were isolated from a soluble fiber extract with determined anti-constipation activity: a 2.04 kDa neutral fraction (SSP-1) contained 99.29% glucose, and a 41.66 kDa acidic fraction (SSP-2) contained 63.85% uronic acid. After mice were given loperamide for 14 d to induce constipation, the GI transit rate increased significantly in the SSP-1 group (*p* < 0.05) but not in the SSP-2 group. The stool weight in the SSP-2 group was significantly higher than that in SSP-1 (383.60 mg vs. 226.23 mg) (*p* < 0.05). Both SSP-1 and SSP-2 groups had significantly increased serum gastrin and motilin levels (*p* < 0.05) and changes in their fecal short-chain fatty acid (SCFA) profiles, while SSP-1 showed better fermentation properties than SSP-2 in terms of statistically higher fecal contents of acetic acid and total SCFAs (*p* < 0.05). Bioinformatic analysis indicated that SSP-1 upregulated bacteria such as *Oscillibacter* to improve SCFA metabolism and stimulate GI hormone secretion, while SSP-2 had less influence on the gut microbiota. These results suggest that the neutral polysaccharide with superior GI fermentation properties exerted beneficial effects on constipation, while the less fermentable pectic fraction might act as a stool-bulking agent.

## 1. Introduction

Constipation is the most frequently reported gastrointestinal (GI) disorder and is characterized by symptoms including infrequent bowel movements, hard consistency of stools, excessive straining, the sensation of incomplete evacuation, and abdominal bloating [1]. The occurrence of constipation is a detriment to the quality of life in multiple aspects, including restrictions on physical functioning, body pain, and also psychological distress. Long-term constipation may contribute to multiple serious maladies, including colorectal cancer, irritable bowel syndrome, and other GI diseases, or even death [2]. Based on epidemiological studies reported in the past three decades, the global prevalence of constipation is estimated to be 10.1% according to the Rome IV criteria [3], with female gender, older age, lower socioeconomic status, less physical activity, depressed psychological status, and physical abuse being identified as common risk factors [4,5]. Regardless of the etiologies, laxatives are the mainstay of pharmacological treatment for potential long-term therapy in constipation patients [6]. However, the quick alleviative effects of pharmacological treatments on constipation are also combined with undesirable side effects, such as diarrhea, nausea, dehydration, and drug dependence [7]. Thus, more effective and safer intervention strategies are needed.

In recent decades, dietary regulation with dietary fiber (DF) or polysaccharide products to treat constipation has received public attention. DF is defined as indigestible carbohydrate (CHO) polymers with a degree of polymerization ≥ 10, according to the Codex Alimentarius Commission, and mainly includes natural or synthetic polysaccharides (cellulose, hemicellulose, pectin, β-glucans, and other CHO polymers), oligosaccharides, resistant starch, and resistant dextrin [8]. For instance, psyllium is a typical laxative DF that is already approved by the Food and Drug Administration (https://www.fda.gov/, accessed on 1 July 2023) and the European Food Safety Authority (https://www.efsa.europa.eu/, accessed on 1 July 2023). In addition, current research shows that polysaccharides from bamboo shavings [9], *Holothuria leucospilota* [10], *Spirulina platensis* [11], *Atractylodis macrocephala* [12], and *Platycodon grandiflorum* [13] also have potential for alleviating constipation. 

The main mechanism by which DF allays constipation is related to its good hydration properties, which help with intestinal peristalsis and increase stool moisture and volume [14]. However, the GI fermentation of DF is also considered an important factor in relieving constipation symptoms. Indigestible fibers can be completely or partially fermented by the gut microbiota in the large intestine, thereby improving intestinal microecology and prompting the production of beneficial metabolites such as short-chain fatty acids (SCFAs) [15]. These organic acids containing 1−6 carbon atoms (e.g., acetic acid, propionic acid, butyric acid, valeric acid) play an essential role in GI transit and defecation. Wang et al. [16] reported that constipated mice treated with resistant acylated or butylated starches presented an increased abundance of acetic acid- or butyric acid-producing bacteria and an enhanced production of acetic acid and butyric acid, which were associated with accelerated intestinal transit and increased stool moisture. Moreover, GI fermentation products of DF also prompt the secretion of GI hormones related to appetite, digestion, and GI motility [17,18]. Lan et al. [19] observed that the motilin (MTL, an excitatory GI hormone) level was reduced in diphenoxylate-induced constipated rats, while inulin and isomalto-oligosaccharide administration alleviated constipation and reestablished the MTL level, as well as increased the SCFA content within the colon. Notably, DF with different compositions and structures may differ greatly in physicochemical properties and GI fermentation performance, which results in divergent responses to constipation interventions. Therefore, it is important to explore the relationship between the structural and fermentative characteristics of DF and its laxative effects to pursue an effective and easy-to-implement therapy for alleviating constipation.

Sweet potato (*Ipomoea batatas* [L.] Lam.) is one of the most widely consumed crops globally and has higher DF content than typical staple foods, such as rice and wheat flour [20]. In general, sweet potato is regarded as a healthy food for GI disorders, e.g., constipation, diarrhea, and inflammatory bowel disease [21]. In our previous study, a soluble fiber composite (SDF-S) was extracted from steamed sweet potato, and it presented superior activity to inulin in promoting *Lactobacillus* spp. proliferation and fermentation in vitro [22]. In our recent preliminary experiment, it was also shown to alleviate loperamide-induced constipation in mice at a dose of 400 mg/(kg·bw·d) (as shown in Appendix A). In the present study, two polysaccharide components with different structures were isolated from SDF-S and were characterized in terms of their chemical composition, molecular weight (Mw), monosaccharide profile, and Fourier transform infrared spectroscopy (FT-IR) spectrum. Their effects on constipation were evaluated by determining the GI motility, stool parameters, intestinal histology, and GI hormone levels in constipated mice. Meanwhile, the fecal SCFA profile and gut microbiota were also analyzed for a better understanding of the fermentation characteristics of the two polysaccharide components. This study is important for understanding the relationship between the fermentation characteristics of polysaccharides and their therapeutic effects on constipation and may be helpful for the selection of polysaccharide laxatives.

## 2. Materials and Methods

### 2.1. Materials and Chemicals

The preparation of SDF-S was conducted using the method that we previously reported [22]. Briefly, 20 min steamed sweet potato was freeze-dried, powdered, and extracted with hot water (40 °C) with the assistance of ultrasonic treatment. After removing starch and protein components with Taka-diastase, amyloglucosidase, and pancreatin, the supernatant of the extract was collected by centrifugation, concentrated, and precipitated with ethanol. The sediment was collected, freeze-dried, and denoted as SDF-S. The lot of SDF-S used in this study was composed of 91.86% glucose, with an average molecular weight of 5.32 kDa. The detailed composition and structural information are presented in Appendix B. SDF-S was further separated by using a DEAE Sepharose Fast Flow (DEAE-FF) ion-exchange column, which was purchased from Solarbio Science & Technology Co., Ltd. (Beijing, China).

Monosaccharide standards (L-arabinose, L-fucose, L-rhamnose, D-galactose, D-glucose, D-mannose, D-ribose, D-glucuronic acid, and D-galacturonic acid) and SCFA standards (acetic acid, propionic acid, n-butyric acid, n-valeric acid, isobutyric acid, and isovaleric acid) were purchased from Sigma-Aldrich Co. LLC (Shanghai, China). Loperamide hydrochloride was purchased from Xi’an Janssen Pharmaceutical Ltd. (Xi’an, China). Phenolphthalein was purchased from Aladdin Biochemical Technology Co., Ltd. (Shanghai, China). Gum arabic was purchased from Yonghua Chemical Co., Ltd. (Suzhou, China). Activated carbon was purchased from Macklin Inc (Shanghai, China). Enzyme-linked immunosorbent assay (ELISA) kits to quantify MTL, gastrin (GAS), substance P (SP), vasoactive intestinal peptide (VIP), and somatostatin (SS) levels in serum were purchased from Lengton Biotech Co., Ltd. (Shanghai, China). All other chemicals and reagents used in this study were of analytical grade.

### 2.2. Preparation of Polysaccharide Fractions from SDF-S

DEAE-FF ion-exchange chromatography was employed to isolate polysaccharide fractions (named SSPs) from SDF-S based on the difference in charge. Firstly, SDF-S was dissolved at 20 mg/mL in distilled water and loaded onto a DEAE-FF column. Then, gradient elution was performed by sequentially using distilled water and NaCl solution with gradually increasing concentrations (0.1, 0.3, and 0.5 mol/L) as the eluent at a flow rate of 3.5 mL/min. Each 10.5 mL of the elution was collected, and its carbohydrate content was determined using the phenol–sulfuric acid method [23]. An elution curve was generated according to the number of the collection and its carbohydrate content. Elution fractions belonging to the same peak were combined, lyophilized, and re-dissolved at a concentration of 0.2 g/mL. Then, this solution was lyophilized again after dialysis at 4 °C for 24 h. As a result of these processes, two fractions were obtained from SDF-S, named SSP-1 and SSP-2, respectively.

### 2.3. Characterization of SSPs

#### 2.3.1. Chemical Composition

The contents of total polysaccharide and uronic acid were quantified by using the phenol–sulfuric acid method with D-glucose, as described in reference [23], and the *m*-hydroxybiphenyl method with D-galacturonic acid, as described in reference [24]. 

#### 2.3.2. Monosaccharide Composition

High-performance liquid chromatography (HPLC) was employed to assay the monosaccharide profiles of SSPs. The samples were hydrolyzed into monosaccharides with 4 mol/L trifluoroacetic acid at 110 °C for 5 h and then dried with N_2_ blowing. The hydrolysate residues were mixed with 0.05 mL of 0.5 mol/L methanolic 1-pheny-3-methyl-5-pyrazolone solution and 0.05 mL of 0.3 mol/L NaOH, incubated at 70 °C for 1 h for esterification, and then neutralized by adding 0.05 mL of 0.3 mol/L HCl. Subsequently, the samples were purified by adding 1.5 mL of trichloromethane, mixing thoroughly, allowing them to stand for 20 min, and carefully discarding the organic phase. After purification 3 times, the resulting aqueous phase was filtered with a 0.22 µm filter and then analyzed using an HPLC system (Agilent 1200, Agilent Technologies Inc, Santa Clara, CA, USA) equipped with an Agilent C18 column (4.6 mm × 250 mm × 5 µm). The chromatogram program was conducted with the following parameters: an injection volume of 10 µL for each sample, a column temperature of 25 °C, a UV detection wavelength of 254 nm, phosphate buffer (0.1 mol/L, pH 6.8) and acetonitrile with a volume ratio of 82:18 as the mobile phase, and an elution flow rate of 1.0 mL/min. Additionally, mixed standards containing rhamnose, arabinose, mannose, ribose, glucose, galactose, fucose, glucuronic acid, and galacturonic acid with certain concentrations were treated with the same protocol as the samples and then analyzed using the above chromatographic program. The concentrations of the eight monosaccharides in the SSPs were quantitatively determined using the external standard method.

#### 2.3.3. Molecular Weight 

A gel permeation chromatography system (Agilent 1260 Infinity II MDS, Agilent Technologies Inc, Santa Clara, CA, USA) equipped with a PL aquagel-OH Mixed-H column (300 mm × 7.8 mm) and a refractive index detector was employed to assay the Mw and homogeneity of SSPs. The samples were prepared in 0.1 mol/L NaNO_3_ solution with a concentration of 3 mg/mL and then filtered with a 0.45 µm filter. The chromatographic conditions were as follows: column temperature, 45 °C; mobile phase, 0.1 mol/L NaNO_3_; flow rate, 1.0 mL/min; injection volume, 50 µL; refractive index increment (dn/dc) value, 0.138 mL/g.

#### 2.3.4. Fourier Transform Infrared Spectroscopy

An FT-IR spectrophotometer (Spectrum Two^TM^, PerkinElmer Inc, Waltham, MA, USA) was employed to analyze the chemical bonds and functional groups of SSPs. Two milligrams of dried sample was ground with 100 mg of KBr into a pellet and scanned in a range of 4000–400 cm^−1^.

### 2.4. Animals and Experimental Design

Eighty male Institute of Cancer Research (ICR) mice were purchased from Zhejiang Vital River Laboratory Animal Technology Co., Ltd. (Jiaxing, China; animal license number: SCXK (ZHE) 2019-0001). After being conditioned for 7 d, the eighty male ICR mice were randomly divided into 5 groups, with sixteen mice in each group. These groups were treated as follows: (1) normal control group (NC), saline; (2) constipation model group (MC), saline + 10 mg/(kg·bw·d) loperamide; (3) positive control group (PC), 10 mg/(kg·bw·d) loperamide + 70 mg/(kg·bw·d) phenolphthalein; (4) SSP-1 group (SSP-1), 10 mg/(kg·bw·d) loperamide + 400 mg/(kg·bw·d) SSP-1; (5) SSP-2 group (SSP-2), 10 mg/(kg·bw·d) loperamide + 400 mg/(kg·bw·d) SSP-2. After a 14 d treatment, mice underwent a GI transit test and defecation function test according to the protocols described below. 

During the experiments, all mice were housed in a specific pathogen-free laboratory at the Laboratory Animal Center of Zhejiang Academy of Agricultural Sciences (Hangzhou, China; facility license number: SYXK (ZHE) 2020-0022). Within the laboratory, the mice were maintained at a temperature of 22 ± 2 °C, a relative humidity of 55 ± 5%, and 12/12 h of automatic lighting (8:00 to 20:00 every day). Except for experimental practices, all of the mice were fed a commercial diet based on AIN-93 M and had free access to drinking water. Body weights and food intake were recorded daily. 

#### 2.4.1. GI Transit Test

The GI transit test was conducted according to the procedure described by Luo et al. [25], and an activated carbon meal solution was prepared according to the method reported by Wang et al. [26]. After the 14 d treatment, eight mice were randomly selected from each group and were fasted for 16 h but had free access to water. Then, the mice were orally administered 0.2 mL of the activated carbon meal solution. Thirty minutes later, the mice were sacrificed by cervical dislocation and dissected to obtain the entire small intestine and cecum contents. The total length of the small intestine and the distance covered by the activated carbon meal were measured. The GI transit rate was calculated using Equation (1):

(1) $$\mathrm{GI}\;\mathrm{transit}\;\mathrm{rate}\;(\%)=\frac{\mathrm{distance}\;\mathrm{covered}\;\mathrm{by}\;\mathrm{activated}\;\mathrm{carbon}\;\mathrm{meal}\;(\mathrm{cm})}{\mathrm{total}\;\mathrm{length}\;\mathrm{of}\;\mathrm{the}\;\mathrm{small}\;\mathrm{intestine}\;(\mathrm{cm})}\;\times \;100$$

Additionally, before intestine dissection, blood samples were collected from the orbits, and the separated serum was used for GI hormone assays. After measuring the intestine’s length, a piece of small intestine tissue about 0.5 cm above the cecum was dissected and fixed in 4% paraformaldehyde for histological observation, and the cecum contents were collected, sealed, and placed on dry ice immediately for gut microbiota analysis.

#### 2.4.2. Defecation Test

The defecation test was conducted according to the method described by Liu et al. [27] with slight modifications. After the 14 d intervention, the remaining eight mice in each group were fasted for 16 h but were allowed free access to drinking water. Then, the mice were orally administered 0.2 mL of the activated carbon meal solution and placed in clean, empty individual cages with accessible feed and drinking water ad libitum for 6 h. The time of the first black stool was recorded. All of the black stools were counted, collected, and weighed on an electronic balance (ATY224, Shimadzu Corporation, Kyoto, Japan). Then, the stool water content was determined by drying the stools at 60 °C for 6 h and was calculated by Equation (2):

(2) $$\mathrm{stool}\;\mathrm{water}\;\mathrm{content}\;(\%)=\frac{\mathrm{stool}\;\mathrm{wet}\;\mathrm{weight}\;(\mathrm{mg})\;-\;\mathrm{stool}\;\mathrm{dry}\;\mathrm{weight}\;(\mathrm{mg})}{\mathrm{stool}\;\mathrm{wet}\;\mathrm{weight}\;(\mathrm{mg})}\;\times \;100$$

### 2.5. Histological Observation

The fixed tissues underwent graded dehydration, paraffin embedding, sectioning, deparaffinization, clearing, and hematoxylin and eosin (H&E) staining according to Feldman et al.’s method [28], and then they were observed with a digital slide scanner (NanoZoomer S60, Hamamatsu Photonics K.K., Shizuoka, Japan).

### 2.6. Assessment of Serum GI Hormones

The levels of MTL, GAS, SP, VIP, and SS in serum were assayed according to the corresponding ELISA kit instructions. The absorbance values were determined at 450 nm using a microplate reader (SpectraMax 190, Molecular Devices LLC, San Jose, CA, USA).

### 2.7. Assessment of Fecal SCFA profile

The fecal SCFA profile was assayed according to the method reported by Zhao et al. [29], with slight modifications. Briefly, the mice feces were mixed with distilled water at a ratio of 1:9 and centrifuged at 10,000 rpm and 4 °C for 15 min. The supernatants were collected and acidified with crotonic acid–metaphosphoric acid solution (0.6464 g of crotonic acid in 100 mL of 2.5% (*w*/*v*) metaphosphoric acid solution) at −20 °C for 24 h. Then, the acidified supernatant was centrifuged at 12,000 rpm for 5 min, filtered, and loaded for SCFA quantification. A GC system (GC-2010 Plus, Shimadzu Corporation, Kyoto, Japan) was employed for quantification, along with a DB-FFAP column (30 m × 0.53 mm × 5 µm, Agilent Technologies Inc, Santa Clara, CA, USA) and a H_2_ flame ionization detector (FID). The injection volume was 1.0 µL, and N_2_ was used as the gas carrier at a flow rate of 12.0 mL/min. The initial column temperature was 70 °C, then increased to 180 °C at a rate of 15 °C/min, and finally increased to 240 °C at 40 °C/min. The temperature of the injection port and FID was kept at 250 °C. The flow rates of air, H_2_, and N_2_ in the detector were 400.0, 40.0, and 30.0 mL/min, respectively.

### 2.8. Bioinformatic Analysis Based on 16S rDNA Sequencing

All cecum contents were sent to LC-Bio Technology Co., Ltd., Hangzhou, Zhejiang Province, China, for bioinformatic analysis. DNA from different samples was extracted using a CTAB kit according to its instructions, and the purity was tested after concentration. The polymerase chain reaction (PCR) amplification of the V3 and V4 regions of bacterial 16S rDNA genes was carried out with the primers 341F (5′-CCTACGGGNGGCWGCAG-3′) and 805R (5′-GACTACHVGGGTATCTAATCC-3′). The PCR products were purified with AMPure XT beads (Beckman Coulter Inc, Indianapolis, IN, USA) and quantified using Qubit (Thermo Fisher Scientific Inc, Waltham, MA, USA). All quantified amplicons were pooled into a library and paired-end-sequenced on a NovaSeq PE250 platform (Illumina Inc, San Diego, CA, USA).

The reads of each sample were merged using FLASH (version 1.2.7, https://ccb.jhu.edu/software/FLASH/index.shtml, accessed on 1 July 2023) and filtered using fqtrim (version 0.94, http://ccb.jhu.edu/software/fqtrim/, accessed on 1 July 2023) and Vsearch software (version 2.3.4, https://github.com/torognes/vsearch, accessed on 1 July 2023) to obtain high-quality clean tags. After dereplication using the Divisive Amplicon Denoising Algorithm (DADA2), the ASV table and sequences were obtained. Then, the ASV sequences were annotated with the SILVA database (release 138, https://www.arbsilva.de/documentation/release138/, accessed on 1 July 2023) for each representative sequence. Alpha diversity (Chao1, Simpson, Shannon, and Pielou’s evenness) and beta diversity (principal coordinate analysis (PCoA) and nonmetric multidimensional scaling (NMDS) based on weighted UniFrac distance) were calculated and normalized to the same sequences randomly using QIIME2 (version 2019.7, https://qiime2.org/, accessed on 1 July 2023). All statistical analyses were performed using R software (version 3.5.2). The Kruskal–Wallis and Wilcoxon rank sum tests were used for comparisons. All diagrams were generated using the Omicstudio platform (https://www.omicstudio.cn/index, accessed on 1 July 2023).

### 2.9. Data Analysis

For all the experiments, measurements were performed in triplicate. Data were collected from the experiments and were first subjected to the Shapiro–Wilk test to examine the normality. Data with a normal distribution are expressed as mean ± standard deviation (SD), and one-way analysis of variance (ANOVA) followed by the Duncan test was performed to compare the differences among groups. Data with a non-normal distribution are expressed as median ± SD, and the Kruskal–Wallis test was conducted to detect differences by comparing the data distribution between groups. Two-tailed Spearman’s correlation tests were conducted to assess the significance of the relationships among constipation-related parameters, fecal SCFAs, and GI microbiota. All of the analyses were conducted by using IBM SPSS program version 26.0 (IBM SPSS Inc, Chicago, IL, USA), with significance at *p* < 0.05. Figures were generated with OriginPro 2022 (OriginLab Co., Northampton, MA, USA).

## 3. Results and Discussion

### 3.1. Physicochemical Characterization of SSPs

**Figure 1:**
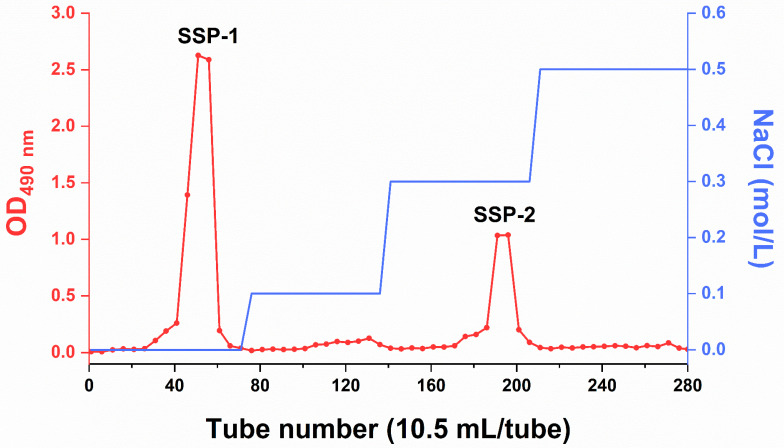
DEAE Sepharose Fast Flow chromatography of SDF-S. SSP-1 and SSP-2 signify, respectively, the neutral and acidic fractions isolated from soluble dietary fiber of steamed sweet potato; OD_490_ = optical density at 490 nm.

According to our previous study [22], SDF-S is a low-Mw, resistant-dextrin-like DF and contains a variety of pectic substances (Appendix B). In the present study, two polysaccharide fractions, i.e., SSP-1 and SSP-2, were separated by using DEAE-FF ion-exchange chromatography, with water and 0.3 mol/L NaCl as the eluents, respectively (Figure 1), suggesting the neutral nature of SSP-1 and the acidic nature of SSP-2. SSP-1 was the main component of SDF-S, with a significantly higher yield of SSP-1 than SSP-2 (61.02 ± 4.62% vs. 12.88 ± 0.74 %) (Table 1). 

**Figure 2:**
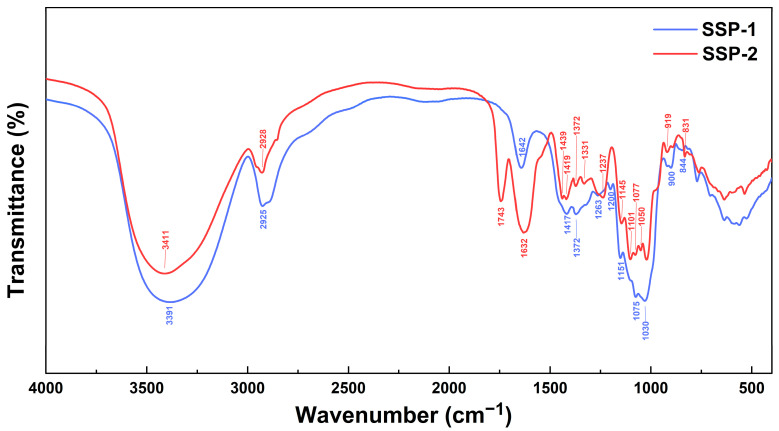
FT-IR spectra of SSPs. SSP-1 and SSP-2, respectively, signify the neutral and acidic fractions isolated from soluble dietary fiber of steamed sweet potato.

The FT-IR spectra of SSP-1 and SSP-2 (Figure 2) presented the typical characteristics of polysaccharides linked by β- and α-glycosidic bonds (900/919 cm^−1^ and 831/844 cm^−1^, respectively) [30]. Additionally, the spectrum of SSP-2 showed a unique, strong band at 1743 cm^−1^, which is attributed to the stretching of esterified C = O [31] and is in accordance with its much higher uronic acid content (Table 1). The monosaccharide profiles (Table 1) suggest that SSP-1 is a neutral polysaccharide with glucose as the predominant constituent (99.49%), while SSP-2 is an acidic polysaccharide characterized by a 63.85% uronic acid content. Two typical acidic monosaccharides, galacturonic acid and glucuronic acid, were determined in SSP-2 with contents of 35.92% and 3.02%, respectively, but were not detected in SSP-1. SSP-1 showed a much lower Mw than SSP-2 (2.04 kDa vs. 41.66 kDa). Based on the above, it is suggested that SSP-1 may be a resistant-dextrin-like component of SDF-S, while SSP-2 may be degraded pectin [32]. 

### 3.2. Effects of SSPs on GI Transit and Defecation Function

The results of GI transit and defecation function tests in constipated mice are presented in Table 2. Compared to the NC group, the MC group presented a significantly decreased GI transit rate (37.93 ± 4.67% vs. 80.19 ± 11.44%) and significantly longer defecation time of the first black stool (156.00 ± 10.15 min vs. 73.50 ± 3.11 min) (*p* < 0.05). In addition, the stool water content, pellet number, and weight in the MC group were also significantly lower (*p* < 0.05), which indicated that loperamide successfully induced constipation in mice. GI transit and defecation function were recovered in constipated mice administered phenolphthalein (the PC group), with no evidence of a significant difference in the GI transit rate or stool parameters being observed between the PC and NC groups, which is in accordance with the previous literature [33].

Both SSP-1 and SSP-2 displayed their potential for relieving constipation while exerting their effects in different aspects. Compared with the MC group, the defecation parameters, including the defecation time, stool water content, and pellet number, were all significantly improved in the SSP-1 and SSP-2 groups (*p* < 0.05). Additionally, the stool weight in the SSP-2 group was also significantly higher than in the MC and SSP-1 groups (*p* < 0.05). The GI transit rate also increased in the two SSP groups compared to the MC group, with a significant difference only being determined between the SSP-1 and NC groups (*p* < 0.05), as shown in Table 2. These results suggest that SSP-2 exerted its effect by improving defecation function, while SSP-1 presented superior effects to SSP-2 by promoting GI motility.

During the experiments, the mice in all groups showed similar weight gain and feeding behavior. No abnormal behavior, disease (other than constipation), or death was observed among mice in any of the groups, suggesting that the different administrations did not disturb the growth of the mice.

### 3.3. Effects of SSPs on Intestinal Histological Morphology

**Figure 3:**
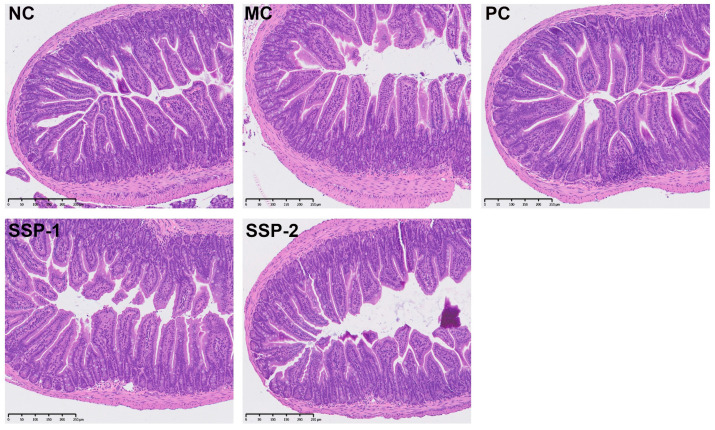
Morphology of mouse small intestine with H&E staining. NC = normal control group; MC = model control group; PC = positive control group; SSP-1 and SSP-2, respectively, signify the groups treated with the two fractions (SSP-1 and SSP-2) isolated from the soluble dietary fiber of steamed sweet potato.

Damage to the small intestinal villi can slow down the peristalsis of the intestine and the transit of excreta, thereby aggravating constipation. The morphology of the mouse small intestine is presented in Figure 3. After H&E staining, the NC group showed a complete intestinal structure with dense and neatly arranged villi, and the epithelial cells had well-separated crypts. However, in the MC group, the villi were disorganized, atrophied, or broken, and the crypts were short, distorted, or missing. Although the small intestinal villi in the PC and SSP-1 groups had some atrophy and rupturing, the villi and crypts in these groups were more intact than those in the CM group, whereas in the SSP-2 group, the villi were severely damaged like those in the MC group, and the crypts were also short, distorted, or missing. These results indicate that treatment with SSP-1 protected or promoted the recovery of the small intestine tissue in constipated mice. 

### 3.4. Effects of SSPs on GI Hormone Levels in Serum

**Figure 4:**
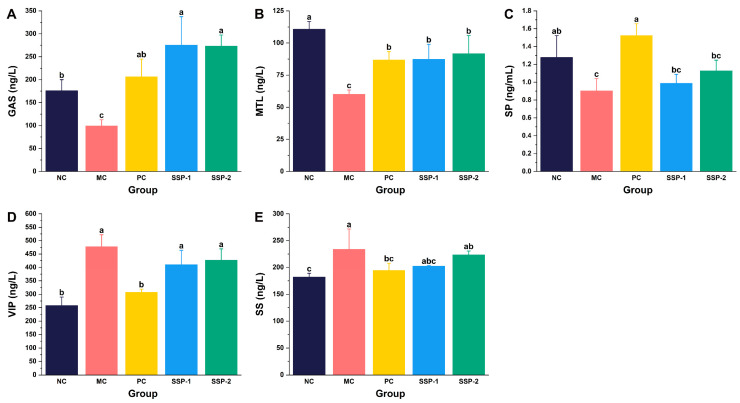
Serum levels of MTL (**A**), GAS (**B**), SP (**C**), VIP (**D**), and SS (**E**) in constipated mice. MTL = motilin; GAS = gastrin; SP = substance P; VIP = vasoactive intestinal peptide; SS = somatostatin. NC = normal control group; MC = model control group; PC = positive control group; SSP-1 and SSP-2, respectively, signify the groups treated with the two fractions (SSP-1 and SSP-2) isolated from the soluble dietary fiber of steamed sweet potato. Different letters between groups indicate significant differences (*p* < 0.05).

The levels of excitatory (GAS, MTL, and SP) and inhibitory GI hormones (VIP and SS) in serum were measured to further explore the mechanisms by which SSPs alleviate constipation. As presented in Figure 4, the GAS, MTL, and SP levels in the MC group were significantly lower than those in the NC group, while the VIP and SS levels were significantly upregulated (*p* < 0.05). The levels of GAS, SP, VIP, and SS in the PC group were not significantly different from those in the NC group (Figure 4 A,C,D,E), while the MTL level was significantly lower than that in the NC group but higher than that in the MC group (*p* < 0.05) (Figure 4B). 

Compared to the MC group, the GAS and MTL levels in the SSP-1 group and SSP-2 group were both significantly higher (*p* < 0.05) (Figure 4A,B). In addition, the GAS level in these groups was even significantly higher than that in the NC group (*p* < 0.05) (Figure 4A). Administering SSP-1 and SSP-2 also increased the SP levels but decreased the VIP and SS levels in constipated mice to some extent, although no statistical difference was detected.

GAS, MTL, SP, VIP, and SS are all essential GI hormones involved in bowel movement. As excitatory hormones, GAS, MTL, and SP can stimulate the secretion of gastric acid and digestive enzymes, thereby promoting GI peristalsis and emptying [34,35,36]. They are usually found at lower levels in constipated patients [37]. In contrast, both VIP and SS inhibit smooth muscle contraction, and SS also exerts broad inhibitory effects on GI hormone secretion (e.g., GAS and SP) [38,39]. In agreement with previous studies [9], the loperamide-treated mice showed remarkable reductions in GAS, MTL, and SP levels, as well as detectable increases in VIP and SS levels, while the up/downregulation of these GI hormones was reversed after treatment with polysaccharides. Thus, the deficiency of excitatory GI hormones and the excessive production of inhibitory GI hormones exert a negative influence on GI motility in mice and lead to constipation. The results demonstrate that both SSP-1 and SSP-2 enhanced the levels of excitatory GI hormones, especially GAS and MTL, thereby improving GI motility in constipated mice.

### 3.5. Effects of SSPs on Fecal SCFA Profile

**Figure 5:**
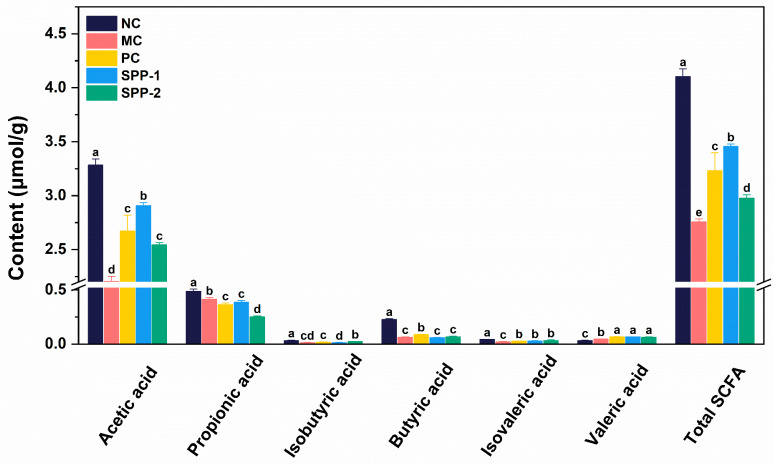
SCFA profile in feces of constipated mice. NC = normal control group; MC = model control group; PC = positive control group; SSP-1 and SSP-2, respectively, signify the groups treated with the two fractions (SSP-1 and SSP-2) isolated from the soluble dietary fiber of steamed sweet potato. Different letters between groups indicate significant differences (*p* < 0.05).

In this study, the contents of acetic acid, propionic acid, isobutyric acid, butyric acid, isovaleric acid, valeric acid, and total SCFAs (the sum of six SCFAs) in mice feces in each group were measured to evaluate the effects of SSPs on gut microbiota metabolites. As shown in Figure 5, except for valeric acid, the contents of all single SCFAs and total SCFAs in the MC group were significantly lower than those in the NC group (*p* < 0.05). SSP treatments improved SCFA production in constipated mice. Compared to the MC group, both the SSP-1 and SSP-2 groups showed significantly higher fecal contents of acetic acid, isovaleric acid, and valeric acid (*p* < 0.05). Additionally, the acetic acid content in the SSP-1 group was significantly higher than in the SSP-2 group. Conversely, the isobutyric acid content in the SSP-2 group was significantly higher than that in the SSP-1 group (*p* < 0.05). 

The function of SCFAs in the intestinal tract mainly includes stimulating peristalsis, promoting fluid secretion, increasing osmotic pressure, and protecting the mucosal barrier, thus performing a vital role in alleviating constipation [40,41]. Therefore, we further analyzed the correlation between SCFAs and other constipation-related parameters. As presented in Table 3, the acetic acid content was positively related to the GI transit rate, stool water content, and serum MTL level but was negatively related to the serum VIP level, defecation time, and SS level (*p* < 0.05). Butyric acid was found to be positively related to the MTL level and SP level but was negatively related to the VIP and SS levels (*p* < 0.05). Positive correlations were found between the fecal isobutyric acid content and the stool weight and MTL level, and a negative correlation was observed between the fecal isobutyric acid content and the serum VIP level (*p* < 0.05). Fecal isovaleric acid was also positively correlated with the stool pellet number, stool weight, stool water content, GI transit rate, and serum MTL level, while it was negatively correlated with the VIP level (*p* < 0.05). Additionally, a positive correlation between fecal valeric acid and GAS and a negative correlation between fecal propionic acid and GAS were detected (*p* < 0.05). 

SCFAs are the major metabolites of indigestible carbohydrates fermented by the gut microbiota. Differences in fecal SCFA contents between the SSP-1 group and the SSP-2 group indicated their different fermentation characteristics, which also accounted for their different capacities for alleviating constipation in mice. The fermentation efficiency of neutral polysaccharides is usually higher than that of pectic polysaccharides, because few bacteria in the intestine can produce sufficient pectin hydrolases [42]. In agreement with existing evidence, the neutral polysaccharide, SSP-1, in this study produced more SCFAs in GI fermentation than the pectic fraction, SSP-2. As a result, the increased SCFAs, especially acetic acid, improved the osmotic pressure of the intestinal tract, promoted intestinal peristalsis, and increased the water content of intestinal contents [43], which explains the higher GI transit rate in the SSP-1 group compared to that in the SSP-2 group. 

### 3.6. Effects of SSPs on the Gut Microbiota 

#### 3.6.1. Effects of SSPs on the Gut Microbial Diversity

**Figure 6:**
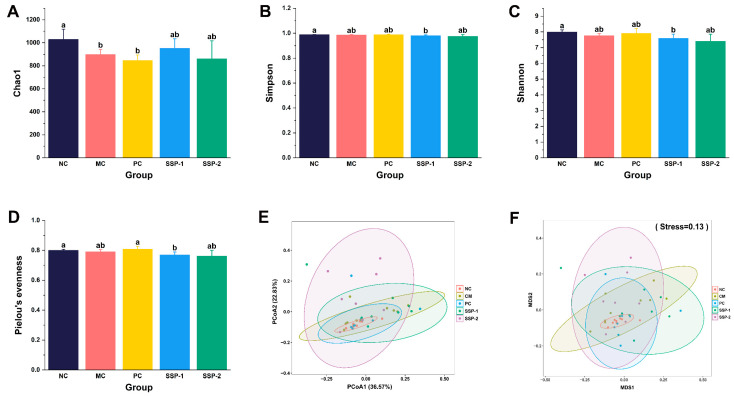
Phylogenetic diversity analysis of gut microbiota in constipated mice. (**A**–**D**) Alpha-diversity analysis indexes include Chao1, Simpson, Shannon, and Pielou’s evenness. (**E**,**F**) Beta-diversity analysis indexes include principal coordinate analysis (PCoA) and nonmetric multidimensional scaling (NMDS) based on weighted UniFrac metric. NC = normal control group; MC = model control group; PC = positive control group; SSP-1 and SSP-2, respectively, signify the groups treated with the two fractions (SSP-1 and SSP-2) isolated from the soluble dietary fiber of steamed sweet potato. Different letters between groups indicate significant differences (*p* < 0.05).

The alpha diversity of the gut microbiota in constipated mice was assessed from several aspects, as depicted in Figure 6. Community richness was characterized by the Chao1 index, community diversity was characterized by the Shannon and Simpson indexes, and community evenness was characterized by Pielou’s evenness index. As can be seen, the Chao1 index significantly decreased in the MC group and PC group compared to the NC group (*p* < 0.05) (Figure 6A), which suggests that loperamide and phenolphthalein administration significantly decreased the richness of the gut microbiota (*p* < 0.05), while the SSP treatments reversed the descent to some extent. In addition, the Simpson, Shannon, and Pielou’s evenness indexes in the SSP-1 group were significantly lower than those in the NC group (*p* < 0.05), while there were no significant differences between the SSP-2 group and other groups (Figure 6B–D). The increase in community richness and the decrease in diversity and evenness indicate that SSP-1 might specifically promote the proliferation of certain bacterial taxa, which also suggests that its fermentation characteristics are different from those of SSP-2. 

The beta diversity was shown by PCoA and NMDS based on the weighted UniFrac metric algorithm. In both plots of PCoA and NMDS (Figure 6E,F), there was evident separation among the SSP-1, SSP-2, and CM groups, indicating the different features of the microbial compositions resulting from the SSP-1 and SSP-2 interventions in constipated mice.

#### 3.6.2. Effects of SSPs on Gut Microbiota Community Structure

**Figure 7:**
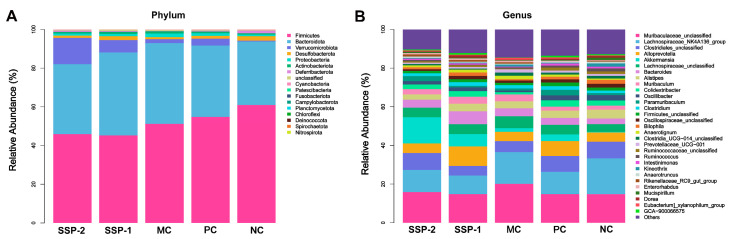
Relative abundance of dominant groups in the gut microbiota of constipated mice at the phylum level (**A**) and genus level (**B**). NC = normal control group; MC = model control group; PC = positive control group; SSP-1 and SSP-2, respectively, signify the groups treated with the two fractions (SSP-1 and SSP-2) isolated from the soluble dietary fiber of steamed sweet potato.

The taxonomic profiles of gut microbiota communities at the phylum level are depicted in Figure 7A. The dominant bacteria in the mouse gut microbiota were Firmicutes and Bacteroidetes, accounting for about 82–93%, followed by Verrucomicrobiota, Desulfobacterota, Proteobacteria, and Actinobacteriota. Compared with the NC group, the relative abundance of Firmicutes in the MC group tended to decrease (60.81% vs. 51.21%), while the relative abundance of Bacteroidetes tended to increase (32.92% vs. 41.71%). After treatment with SSP-1 or SSP-2, the relative abundance of Firmicutes was further reduced (45.15% and 45.90%, respectively), while the relative abundance of Bacteroidetes was increased (42.94% and 36.16%, respectively). In addition, SSP treatments also improved the relative abundance of Verrucomicrobia. 

**Figure 8:**
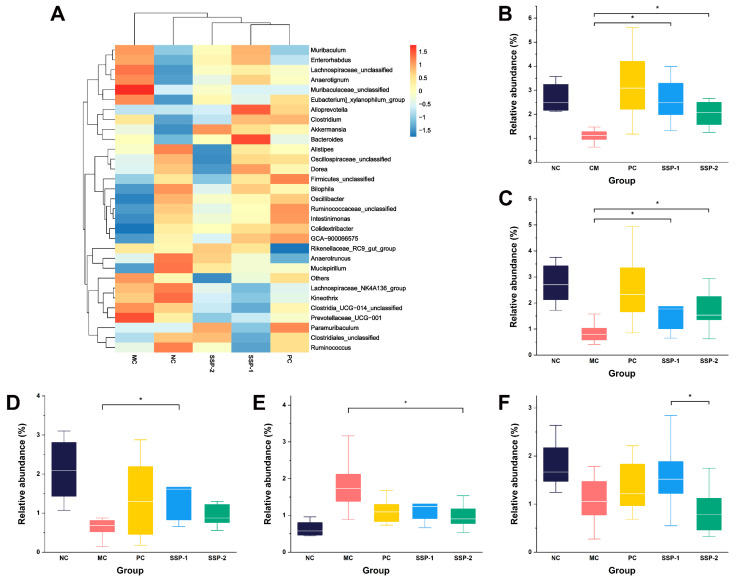
Heatmap comparison between experimental groups at genus level (**A**) and relative abundance of *Colidextribacter* (**B**), *Oscillibacter* (**C**), *Bilophila* (**D**), *Anaerotignum* (**E**), and *Oscillospiraceae unclassified* (**F**) in the gut microbiota of constipated mice. NC = normal control group; MC = model control group; PC = positive control group; SSP-1 and SSP-2, respectively, signify the groups treated with the two fractions (SSP-1 and SSP-2) isolated from the soluble dietary fiber of steamed sweet potato. * Indicates significant differences between MC group and SSP groups (*p* < 0.05).

To further explore the differences in the community between groups, the relative abundance (Figure 7B) and heatmap (Figure 8A) of the dominant bacteria genera (relative abundance ≥ 0.5%) were used to show the differences at the genus level. Compared to the NC group, the MC group had a significantly lower relative abundance of *Oscillospiraceae unclassified*, *Bilophila*, *Ruminococcaceae unclassified*, *Ruminococcus*, *Intestinimonas*, *Mucispirillum*, and *GCA-900066575*, while it had a significantly higher relative abundance of *Anaerotignum* (*p* < 0.05). Compared to the MC group, both the SSP-1 and SSP-2 groups had a significantly higher relative abundance of *Colidextribacter* and *Oscillibacter* (Figure 8B,C) (*p* < 0.05). The significant differences between the SSP-1 group and SSP-2 group at the genus level lay in the relative abundances of *Bilophila*, *Anaerotignum*, and *Oscillospiraceae unclassified*. In the loperamide-induced constipated mice, administering SSP-1 significantly increased the richness of *Bilophila* (Figure 8D) (*p* < 0.05), while SSP-2 gavage significantly decreased the abundance of *Anaerotignum* (Figure 8E) (*p* < 0.05). In addition, the relative abundance of *Oscillospiraceae unclassified* in the SSP-1 group was significantly higher than in the SSP-2 group (*p* < 0.05), though neither was statistically different from the MC group. These results show that constipation reduced the relative abundance of some beneficial or harmless commensal bacteria, while treatment with SSPs could increase the relative abundance of these bacteria.

The community structure of the gut microbiota is an important factor related to the occurrence of constipation [44]. *Oscillibacter*, *Oscillospiraceae unclassified*, and *Colidextribacter* belong to the family Oscillospiraceae, which can utilize simple dietary carbohydrates or host glycans and secrete leptin and butyrate [45]. *Bilophila* is a sulfate-reducing opportunistic bacterial pathogen belonging to the family Desulfovibrionaceae [46]. Although it is suspected to negatively affect host metabolic function, the hydrogen sulfide (H_2_S) it produces might have excitatory effects on GI motility [47]. *Anaerotignum* is a genus of the family Lachnospiraceae. It was reported that *Anaerotignum* could produce beneficial SCFAs, whereas in the study by Zhu et al. [48], its relative abundance was negatively correlated with Na^+^-K^+^-ATP-ase and Ca^2+^-Mg^2+^-ATP-ase in a diarrhea mouse model and might affect energy metabolism in mice. In this study, the constipated model mice also had a higher relative abundance of *Anaerotignum* than normal mice, suggesting its unrevealed role in GI disorder to be further investigated. The results also indicate that the neutral polysaccharide SSP-1 showed a greater impact on the gut microbiota of constipated mice than SSP-2, especially on regulating the beneficial bacterial genera. Although the pectic polysaccharide SSP-2 was less fermentable, its presence in the intestine also inhibited the proliferation of some constipation-related bacterial genera.

#### 3.6.3. Change in Key Phylotypes of the Gut Microbiota

**Figure 9:**
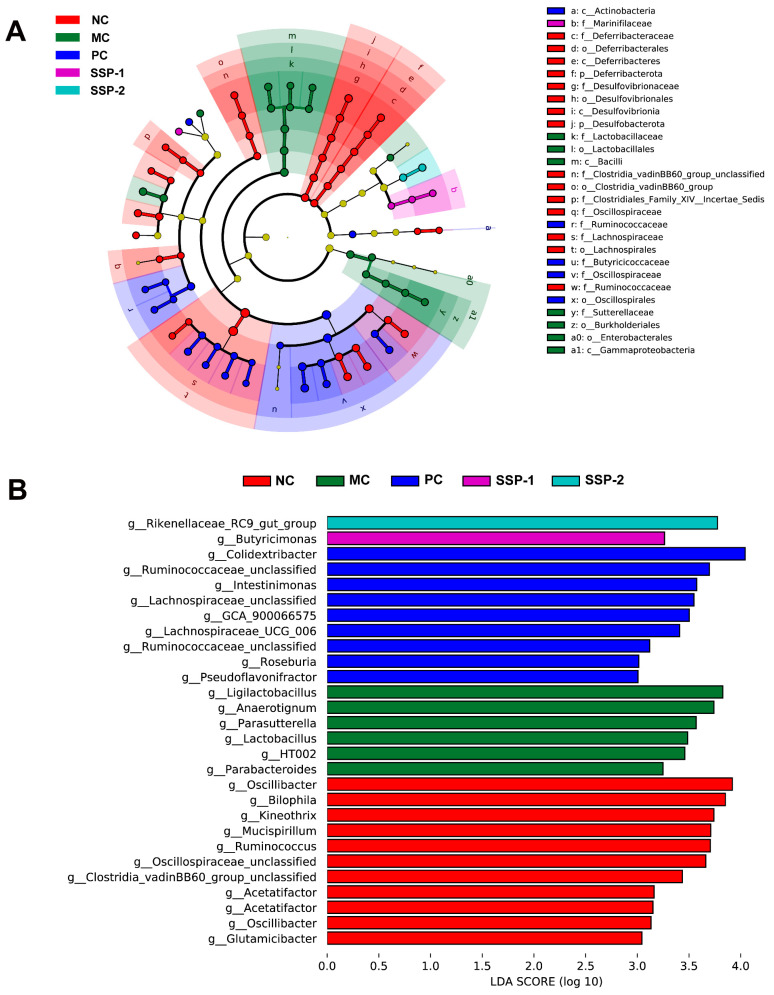
Major categories of the gut microbiota in constipated mice. (**A**) Presentation of the relationship between taxa derived from LEfSe cladogram (the levels are class, order, family, and genus from the inner to outer rings). (**B**) The genus-level biomarker taxa LDA scores (>3) based on LEfSe analysis (the length of the bar means the LDA score). NC = normal control group; MC = model control group; PC = positive control group; SSP-1 and SSP-2, respectively, signify the groups treated with the two fractions (SSP-1 and SSP-2) isolated from the soluble dietary fiber of steamed sweet potato.

An LDA Effect Size (LEfSe) analysis was conducted to compare the specific bacterial taxa associated with constipation for all groups of mice (‘c_’, class; ‘o_’, order; ‘f_’, family; ‘g_’, genus). As depicted in Figure 9A, the seven dominant families in the NC group belonged to Deferribacteraceae, Desulfovibrionaceae, Clostridia vadinBB60 group unclassified, Clostridiales Family XIV Incertae Sedis, Oscillospiraceae, Lachnospiraceae, and Ruminococcaceae. Lactobacillaceae and Sutterellaceae were the most abundant in the MC group. The higher taxonomies from three key families in the PC group were Ruminococcaceae, Butyricicoccaceae, and Oscillospiraceae. The treatment of SSP-1 boosted the family Marinifilaceae in constipated mice. As depicted in Figure 9B, the LDA score (>3) demonstrated that high abundances of *HT002*, *Ligilactobacillus*, *Lactobacillus* (all belonging to the family Lactobacillaceae), *Anaerotignum* (family Lachnospiraceae), *Parasutterella* (family Sutterellaceae), and *Parabacteroides* (family Tannerellaceae) were identified in the MC group, while *Oscillibacter*, *Oscillospiraceae unclassified* (both belonging to the family Oscillospiraceae), *Acetatifactor*, *Kineothrix* (both belonging to the family Lachnospiraceae), *Glutamicibacter* (family Micrococcaceae), *Mucispirillum* (family Deferribacteraceae), *Clostridia vadinBB60 group unclassified* (family Clostridia vadinBB60 group unclassified), *Bilophila* (family Desulfovibrionaceae), and *Ruminococcus* (family Ruminococcaceae) played major roles in the NC group. After the interventions with SSP-1 and SSP-2, the constipated mice had higher abundances of *Butyricimonas* (family Marinifilaceae) and *Rikenellaceae RC9 gut group* (family Rikenellaceae), respectively.

#### 3.6.4. Correlations between the Gut Microbiota and Constipation-Related Biological Parameters

**Figure 10:**
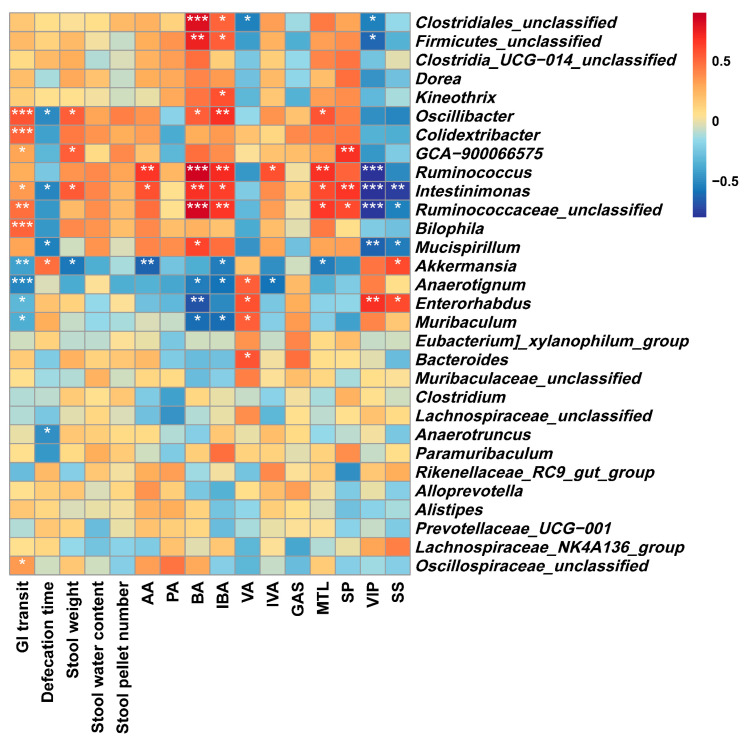
Heatmap of Spearman’s correlations between the gut microbiota and biological parameters (* *p* < 0.05, ** *p* < 0.01, *** *p* < 0.001).

To investigate the association between the gut microbiota and constipation-related biological parameters, Spearman’s correlation analysis was conducted, and the correlation heatmap is presented in Figure 10. Seven bacteria were found to be positively correlated with the GI transit rate (*p* < 0.05), including *Oscillibacter*, *Colidextribacter*, *Intestinimonas*, *Bilophila*, *Ruminococcaceae unclassified*, etc. As discussed in Section 3.6.1, these bacterial genera were the main differences between the SSP treatment groups and the MC group. In addition, there were also four bacteria negatively correlated with GI transit, among which the genus *Anaerotignum* (downregulated in SSP-2 group, Figure 8E) had the highest correlation (*p* < 0.001). The genera *Oscilliabacter*, *Intestinimonas*, *Mucispirillum*, and *Anaerotruncus* were negatively correlated with the defecation time (*p* < 0.05), while they were positively correlated with the other stool parameters, including the water content, weight, and pellet number of stools (*p* < 0.05). These results actually further suggest that the two SSPs act through different mechanisms to alleviate constipation.

Most of the bacteria significantly downregulated in the MC group were positively correlated with SCFA production (*p* < 0.05), especially the production of acetic acid, butyric acid, and isobutyric acid. *Oscillibacter*, as one of the genera upregulated in the SSP treatment groups (Figure 8C), was found to be positively correlated with the SCFA contents in feces, which is also consistent with its metabolic characteristics reported in a previous study [45]. In addition, four genera (*Anaerotignum*, *Bacteroides*, *Enterorhabdus*, and *Muribaculum*) were positively correlated with valeric acid production (*p* < 0.05) and were upregulated in the SSP-1 group, especially for the genus *Bacteroides* (Figure 8A). 

As for the GI hormones, *Ruminococceae unclassified*, *Ruminococcus*, and *Intestinimonas* were positively correlated with excitatory GI hormones (MTL and SP) (*p* < 0.05) and were negatively correlated with inhibitory GI hormones (VIP and SS) (*p* < 0.05). These three bacteria were all downregulated in the MC group. In addition, it was also *Oscillibacter* that showed a significant positive correlation with the serum MTL level (*p* < 0.05). Among these bacteria, *Oscillibacter* or the family Oscillospiraceae seemed to be the key bacteria that were regulated by SSPs, and their metabolites exerted a positive influence on GI transit, defecation, and GI hormone secretion. Moreover, the bacteria specifically regulated by SSP-1 and SSP-2, i.e., *Bilophila* and *Anaerotignum*, presented different correlations with these biological parameters. 

Current therapies for constipation mainly use fiber as a bulking agent, which increases stool moisture and accelerates stool transit [49]. However, emerging research provides noteworthy evidence that GI fermentation can alleviate constipation by producing beneficial metabolites and stimulating neurotransmitters or hormone secretion. Zhang et al. [50] reported that chitosan oligosaccharides attenuated loperamide-induced constipation in mice while modulating the production of bile acids, SCFAs, and tryptophan catabolites by regulating beneficial bacteria such as *Faecalibacterium*, *Lactobacillus*, *Bacteroides*, and *Alistipes*. They also reported that chitosan oligosaccharides failed to improve these constipated mice with gut microbiota depletion. Liang et al. [51] reported that low-Mw chitosan (1 kDa) showed better laxative effects on constipated mice than high-Mw chitosan in terms of GI transit and defecation frequency. It also significantly upregulated the levels of *Bacteroides* and *Parabacteroides,* while it downregulated the level of *Desulfovibrio*, as well as promoted the production of SCFAs. These studies demonstrated that the role of the gut microbiota and its metabolites in alleviating constipation was more dominant than the bulking or water-binding capacity of fiber, and these easily fermentable fibers could help the gut microbiota produce more beneficial metabolites than less fermentable ones.

In the present study, as a low-Mw, resistant-dextrin-like polysaccharide, SSP-1 promoted the proliferation of bacteria of the family Oscillospiraceae or Ruminococceae. These beneficial bacteria likely preferred utilizing fermentable carbohydrates with a single composition or simple structure [52,53]. The abundant metabolites that these bacteria produced exerted positive effects on GI hormone regulation and GI motility. In contrast, the relieving effect of the pectic polysaccharide SSP-2 on constipation symptoms might be attributed to bulking stool. The hydration characteristics of its residue from GI fermentation helped improve the defecation function in constipated mice. It is worthy to note that the fermentation of polysaccharides by the GI microbiota leads to the production of gases, including hydrogen (H_2_) and carbon dioxide, among others. Excessive gas production can cause adverse effects such as discomfort, flatus, and bloating [4]. Therefore, it is crucial to further evaluate the gas production of these two SSPs resulting from GI microbiota fermentation, so as to fully assess their efficiencies on the treatment of constipation-related disadvantages.

## 4. Conclusions

This study compared the anti-constipation effects of two polysaccharide fractions, a neutral, resistant-dextrin-like polysaccharide and an acidic, pectin-derived polysaccharide, which were simultaneously isolated from steamed sweet potato extracts. The neutral fraction was superior to the acidic fraction in terms of the significant improvement in GI motility and better histological morphology of small intestinal villi, while the acidic fraction showed a better stool-bulking effect. The neutral fraction improved GI homeostasis by increasing the richness of beneficial bacteria such as *Oscillibacter* and producing more fecal acetic acid and total SCFAs. In contrast, the acidic fraction presented an inferior effect on regulating the GI microbiota. It could be concluded that the GI fermentation of polysaccharides played a more important role than its stool-bulking effect in alleviating constipation, and the production of acetic acid and total SCFAs accounted for the recovery of GI motility and defecation function in constipated mice. GI fermentability should be emphasized when a polysaccharide is considered as a potential therapeutic agent. While the gas production resulted from polysaccharide fermentation should be evaluated prudently, as far as the possible side effect is concerned.

## Figures and Tables

**Table 1:** Chemical composition of SSPs.

Items	SSP-1	SSP-2
Yield (%)	61.02 ± 4.62 ^a^	12.88 ± 0.74 ^b^
Neutral carbohydrate (%)	81.79 ± 0.87 ^a^	34.63 ± 1.29 ^b^
Uronic acid (%)	3.75 ± 0.32 ^b^	63.85 ± 0.42 ^a^
Monosaccharide (mol%)		
D-Glucose	99.29	11.34
D-Galactose	0.42	26.25
D-Mannose	0.19	4.25
L-Arabinose	0.08	8.57
L-Rhamnose	0.02	9.70
D-Ribose	0.01	0.29
L-Fucose	N.D.	0.66
D-Galacturonic acid	N.D.	35.92
D-Glucuronic acid	N.D.	3.02
Mw (kDa)	2.04	41.66
Mw/Mn	1.24	1.09

Note: The results are expressed as means ± SD. Different letters in the same row show significant differences (*p* < 0.05). SSP-1 and SSP-2 signify, respectively, the neutral and acidic fractions isolated from soluble dietary fiber of steamed sweet potato. N.D. = not detected; Mw = molecular weight; Mn = number-averaged molecular weight.

**Table 2:** Effects of SSPs on GI transit and stool parameters in constipated mice.

Index	Group				
	NC	MC	PC	SSP-1	SSP-2
Gastrointestinal transit rate (%)	80.19 ± 11.44 ^a^	37.93 ± 4.67 ^c^	75.85 ± 16.53 ^a^	59.20 ± 9.23 ^b^	48.50 ± 7.79 ^bc^
First black stool defecation time (min)	73.50 ± 3.11 ^b^	156.00 ± 10.15 ^a^	79.75 ± 10.14 ^b^	81.25 ± 14.86 ^b^	82.50 ± 9.18 ^b^
Stool water content (%)	67.07 ± 6.36 ^a^	49.57 ± 7.28 ^b^	60.50 ± 8.87 ^a^	64.63 ± 2.65 ^a^	64.79 ± 2.77 ^a^
Stool pellet number (ea)	18.67 ± 8.39 ^ab^	4.00 ± 2.65 ^c^	15.33 ± 4.51 ^b^	17.67 ± 3.06 ^ab^	25.00 ± 3.74 ^a^
Stool weight (mg)	428.00 ± 35.64 ^a^	109.53 ± 88.65 ^d^	284.33 ± 62.88 ^bc^	226.23 ± 42.32 ^cd^	383.60 ± 126.79 ^ab^

Note: The results are expressed as means ± SD. Different letters show significant differences in a row (*p* < 0.05). NC = normal control group; MC = model control group; PC = positive control group; SSP-1 and SSP-2, respectively, signify the groups treated with the two fractions (SSP-1 and SSP-2) isolated from the soluble dietary fiber of steamed sweet potato; ea means the stool pellet number of one single mouse.

**Table 3:** Correlations among SCFAs, GI transit, stool parameters, and GI hormones.

Indicators	Acetic Acid	Propionic Acid	Butyric Acid	Isobutyric Acid	Valeric Acid	Isovaleric Acid
Gastrointestinal transit rate	0.768 *	0.264	0.400	0.504	0.111	0.750 *
Stool pellet number	0.309	−0.499	0.211	0.409	0.116	0.570 *
Defecation time	−0.552 *	0.077	−0.247	−0.382	−0.241	−0.502
Stool weight	0.236	−0.389	0.393	0.514 *	0.050	0.554 *
Stool water content	0.657 *	0.054	0.289	0.439	0.007	0.543 *
GAS	0.318	−0.650 *	−0.071	−0.061	0.604 *	0.268
MTL	0.814 *	0.114	0.679 *	0.614 *	−0.061	0.704 *
SP	0.324	−0.147	0.574 *	0.390	0.225	0.357
VIP	−0.717 *	−0.311	−0.794 *	−0.519 *	0.249	−0.524 *
SS	−0.579 *	−0.400	−0.575 *	−0.221	0.311	−0.271

Note: The asterisk (*) denotes statistically significant correlation coefficients (*p* < 0.05). MTL = motilin; GAS = gastrin; SP = substance P; VIP = vasoactive intestinal peptide; SS = somatostatin.

**Figure A1:**
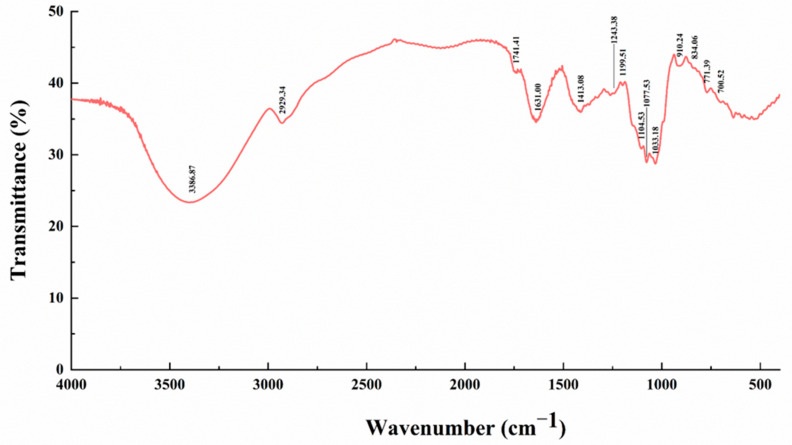
FT-IR spectra of SDF-S.

## Data Availability

All raw data supporting the reported results are available from the authors on request.

## Appendix A. The Pre-Experimental Design of the Effective Dose of SDF-S in Alleviating Constipation in Mice

### Appendix A.1. Methods

To evaluate the improvement of constipation in mice with the administration of SDF-S, sixty male ICR mice were acclimated for 14 d and then randomly divided into 6 groups, which were the normal control (NC), model control (MC), positive control (PC), low-dose (LD), middle-dose (MD), and high-dose (HD) groups. Except for the NC group, the mice in the other groups were given loperamide by gavage with a dose of 10 mg/(kg·bw·d) to induce constipation. During the same period, mice in the PC group were given phenolphthalein at a dose of 70 mg/(kg·bw·d), which was the same as in previous research. The mice in the LD, MD, and HD groups were given SDF-S at doses of 200, 400, and 800 mg/(kg·bw·d), respectively, while the mice in the NC and MC groups were given saline. After 14 d, the mice were subjected to the GI transit test to determine the effective dose.

### Appendix A.2. Results

As shown in Table A2, the body weight of mice on the first day of experiments was not significantly different among any of the groups, suggesting that the initial status of mice in all groups was identical. The body weight gains of mice in the SDF groups were all significantly lower than those of the mice in the NC group (*p* < 0.05) after 14 d of experiments. The daily feed intake of constipated mice was lower than that of normal mice, especially in the MC, LD, and MD groups, whose daily feed intake was significantly lower than that of the NC group (*p* < 0.05). The differences in body weight changes and daily feed intake suggest that SDF-S might regulate the appetite of constipated mice and promote their weight reduction. However, no abnormal behavior, disease (other than constipation), or death was observed among mice in any of the groups, suggesting that the differences in feeding behavior due to different administrations might not interfere with the following assessments.

The GI transit rate in the MC group was significantly lower than that of the NC group (*p* < 0.05) and was the lowest among all groups, which indicated that loperamide had successfully delayed GI peristalsis. In the PC group, the GI transit rate was only significantly lower than that in the NC group (*p* < 0.05), suggesting that the treatment with phenolphthalein improved GI motility in constipated mice. The GI transit rates of the SDF-S groups were all higher than that of the CM group and were in the order of HD group > MD group > LD group. The results indicate that SDF-S could alleviate constipation and was dose-dependent. Among the low, medium, and high doses of SDF-S, only the constipated mice treated with a high dose of SDF-S (HD group) showed a significantly higher GI transit rate than the mice in the MC group (47.71% vs. 33.87%) (*p* < 0.05), and they showed no significant differences from the PC group (*p* > 0.05). Thus, the effective dose of SDF-S to alleviate constipation in mice was 400 mg/(kg·bw·d) and was adopted to compare the laxative effects of SSP-1 and SSP-2.

**Table A1:** Effects of different doses of SDF-S on GI transit in constipated mice.

Group	Body Weight-0 d (g)	Body Weight-14 d (g)	Daily Feed Intake (g)	Gastrointestinal Transit Rate (%)
NC	30.98 ± 1.09 ^a^	34.11 ± 1.49 ^a^	4.27 ± 0.31 ^a^	75.06 ± 5.32 ^a^
MC	30.75 ± 1.47 ^a^	33.45 ± 2.42 ^ab^	3.64 ± 0.35 ^bc^	33.87 ± 8.46 ^d^
PC	30.64 ± 1.89 ^a^	33.28 ± 1.79 ^abc^	4.05 ± 0.33 ^a^	49.36 ± 9.45 ^b^
LD	30.04 ± 2.74 ^a^	31.28 ± 2.29 ^c^	3.44 ± 0.34 ^c^	37.02 ± 11.05 ^d^
MD	29.60 ± 1.28 ^a^	31.39 ± 1.79 ^bc^	3.76 ± 0.26 ^b^	40.58 ± 8.13 ^cd^
HD	30.49 ± 1.88 ^a^	32.00 ± 2.36 ^bc^	4.08 ± 0.31 ^a^	47.71 ± 8.65 ^bc^

Note: The results are expressed as means ± SD. Different letters show significant differences in a column (*p* < 0.05). NC = normal control; MC = model control; PC = positive control; LD = low dose; MD = middle dose; HD = high dose; GI = gastrointestinal.

## Appendix B. Characterization of SDF-S

**Table A2:** Chemical composition of SDF-S.

Item	SDF-S
Neutral carbohydrate (%)	59.31 ± 0.73
Uronic acid (%)	25.36 ± 0.48
Total carbohydrate (%)	84.68 ± 0.64
Iodine reactions	Negative
Mw (kDa)	5.32
Mw/Mn	1.605
Monosaccharide composition (mol%)	
Glc	91.86
Gal	3.15
Ara	1.07
Rha	0.65
Man	0.42
Rib	0.11
Fuc	0.13
GlcA	0.14
GalA	2.48

The data are expressed as mean ± SD. Data with different letters within the same row are significantly different at *p* < 0.05. SDF-S signify the soluble dietary fiber isolated from steamed sweet potato; Glc, glucose; Gal, galactose; Ara, arabinose; Rha, rhamnose; Man, mannose; Rib, ribose; Fuc, fucose; GlcA, glucuronic acid; GalA, galacturonic acid. Mw = molecular weight; Mn = number-averaged molecular weight.
